# Effect of Heat Stimulation on Circulating Irisin in Humans

**DOI:** 10.3389/fphys.2021.675377

**Published:** 2021-06-28

**Authors:** Tae-Hwan Park, Hye-Jin Lee, Jeong-Beom Lee

**Affiliations:** ^1^College of Medicine, Soonchunhyang University, Cheonan, South Korea; ^2^Department of Physiology, College of Medicine, Soonchunhyang University, Cheonan, South Korea

**Keywords:** irisin, hyperthermia, oxidative stress, cortisol, CK, LDH

## Abstract

High temperatures lead to oxidative stress. The aim of the study was to determine whether heat stimulation-induced hyperthermia can increase the level of circulating irisin. Twenty-one healthy female subjects (age, 26.3 ± 2.71 years; height, 162.1 ± 3.15 cm; weight, 54.2 ± 3.86 kg; and body surface area, 1.57 ± 0.11 m^2^) not taking contraceptives participated in this study. All experiments were performed individually for each participant when they were in the early proliferative menstrual phase. In an automated climate chamber (25 ± 0.5°C), the heat load was applied via half-body immersion into a hot water bath (42 ± 0.5°C). Five-minutes break was provided every after 5 min of immersion and the total passive heating time was 30 min. Tympanic temperature (T_t__y_) and skin temperature (T_s_) were measured. Mean body temperature (mT_b_) was calculated. Blood samples were collected before and immediately after immersion. Levels of irisin, cortisol, creatine kinase (CK), and lactate dehydrogenase (LDH) were analyzed. T_ty_, mT_b_ and serum irisin levels increased after hot water immersion. The blood levels of cortisol, CK, and LDH were also elevated after hot water immersion. Heat stimulation might increase the levels of circulating irisin in humans in response to oxidative stress.

## Introduction

Irisin is a newly discovered myokine: proteolytic fibronectin type III domain containing 5 (FNDC5)-cleaved product. It induces browning of subcutaneous adipose tissues via elevation of uncoupling protein 1 and leads to thermogenesis and metabolic improvement (Bostrom et al., 2012).

Circulating levels of irisin increase transiently during acute exercise, and irisin concentrations are positively correlated with the intensity of exercise (Bostrom et al., 2012; Huh et al., 2014a; Huh and Mantzoros, 2015). However, such an effect was less pronounced in previous studies. Some studies failed to confirm the increase in irisin levels after chronic training (Huh et al., 2014b; Kraemer et al., 2014; Singhal et al., 2014). Irisin has been shown to increase temporarily after exercise, but not sustained in the long term (Huh et al., 2014b; Kraemer et al., 2014; Singhal et al., 2014).

Samy et al. (2015), recently reported that irisin concentration was related to oxidative stress and muscle damage, and suggested that unlike chronic exercise, acute exercise may increase oxidative stress significantly, resulting in irisin secretion from the muscle. However, whether different types of oxidative stress contribute to higher levels of irisin are unknown.

The aim of the present study is to evaluate whether heat stimulation-induced hyperthermia can increase circulating irisin levels after half-body immersion in hot water. High temperatures lead to oxidative stress (Portner, 2001; Portner and Knust, 2007) and elicit whole-body responses, involving skeletal and cardiac muscle tissues. In addition to irisin, we also analyzed blood creatine kinase (CK) and lactate dehydrogenase (LDH) levels as indicators of heat stimulation-induced oxidative damage.

## Materials and Methods

### Subjects

Twenty-one healthy female subjects, who lived all of their lives in the city of Cheonan (Chungnam), South Korea, voluntarily participated in this study. Cheonanis located in southwestern Korea (126°52′N, 33.38′E) and extends northeast (130°4′N, 43.0′E). The mean annual ambient temperature during October 2019 to March 2020 was 5.3°C with 73.0% relative humidity. All subjects were Korean women who lived in Cheonan for 3 months before the experiment and during the experimental period (January 2020 to March 2020).

The subjects (*n* = 21, age, 26.3 ± 2.71 years; height, 162.1 ± 3.15 cm; weight, 54.2 ± 3.86 kg; body surface area, 1.57 ± 0.13 m^2^; body mass index, 25.8 ± 3.45; % fat, 20.6 ± 3.85) were not taking contraceptives. Body surface area was calculated according to the Du Bois formula (Du Bois and Du Bois, 1916). Body fat percentage was measured through the bio-impedance method (InBody 770, Seoul, Korea).

Progesterone released from the corpus luteum after ovulation affects the hypothalamus, raising basal body temperature. Therefore, inspired by a preceding research, we investigated self-reports of our subjects about the menstrual cycle and performed the protocol individually for each participant when they were in the early proliferative menstrual phase, 3–7 days after the start of menstrual bleeding (Stachenfeld et al., 2000; Baker et al., 2020).

The subjects fasted for 6 h before the test and were instructed to refrain from consuming alcohol or medication 48 h before the test. Each subject was thoroughly briefed with the purpose of the study, experimental procedures, and potential risks. All procedures complied with the 2013 Helsinki Declaration of the World Medical Association from the Institutional Review Board on Human Subjects Research and Ethics Committees, Soonchunhyang University (No. 2016R1D1A3B02015394), Cheonan, Korea.

### Measurement and Experimental Procedure

Tests were performed in a climate chamber under the following conditions: temperature, 25.0 ± 0.5°C; relative humidity, 60.0 ± 3.0%; air velocity, 1 m/s (Schematic 1). The experiment was conducted between 2 and 5 p.m. to control for the influence of circadian rhythm on the body temperature. The subject sat in a chair in a relaxed posture for 60 min before the start of the main process. The heat load experiment was carried out by immersing half the body (up to waist) into a bath filled with hot water of 42 ± 0.5°C, which was a setting of similar thermal intensity often used in previous studies (Lee et al., 2013; Shin et al., 2013; Lee and Shin, 2017; Lee and Kim, 2018). Since not all our subjects have experienced the heat loading before, half-body immersion in hot water could cause some discomfort. Thus, 5-min breaks in the chamber air temperature were provided every after 5 min of heat loading (Schematic 1). Additionally, a drinkable 500mL of tepid (25.0 ± 0.5°C) water bottle was provided for each subjects to prevent dehydration.

**Schematic 1:**
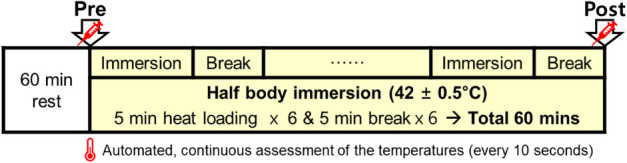
Schematic representation of the experimental protocol condition in air temperature 25.0 ± 0.5°C and relative humidity 60.0 ± 3.0%. Heat stimulation entailed immersing half of the body into a hot water bath (42 ± 0.5°C) for total 30 min and break for total 30 min. Blood sampling was done before immersion (Pre) and immediately after the last break (Post). Tympanic and local skin temperature measurements were conducted continuously at 10-s intervals for 60 min.

We checked whether the subjects had any symptoms of infection including fever, and the bathtub was washed cleanly every time after it was used. This process has been carried out more thoroughly since mid-February, when the COVID-19 epidemic began in Korea. Except for one subject immersing in the bath, all investigators and the other subjects wore masks and periodic ventilation and alcohol disinfection of the chamber were performed.

### Tympanic Temperature and Mean Body Temperature Measurements

Tympanic temperature (T_ty_) was assessed continuously at 10-s intervals for 60 min in the left ear by inserting a thermistor probe (TSK7+1, Songkitopia, Inchen, South Korea) with a small spring (K923, Takara, Yokohama, Japan) connected to a personal computer (CF-T1, Panasonic, Tokyo, Japan) and a data logger (K-720, Technol Seven, Yokohama, Japan) (Lee et al., 2013; Shin et al., 2013; Lee and Shin, 2017; Lee and Kim, 2018). When the thermistor probe contacted the tympanic membrane, the subject felt slight discomfort and could hear a scratching noise. The inner pinna was filled with small cotton balls to fix the probe in the ear (Lee et al., 2013; Shin et al., 2013; Lee and Shin, 2017; Lee and Kim, 2018).

Skin temperatures of the chest (Ts_chest_), upper arm (Ts_arm_), thigh (Ts_thigh_), and leg (Ts_leg_) were measured (continuous assessment, Schematic 1) with a TSK7+1 thermistor probe (Songkitopia, Incheon, South Korea) and a K923 small spring (Takara, Yokohama, Japan) (Lee et al., 2013; Shin et al., 2013; Lee and Shin, 2017; Lee and Kim, 2018). The probe was connected to a CF-T1 personal computer (Panasonic, Tokyo, Japan) and a K-720 data logger (Technol Seven, Yokohama, Japan). Mean skin temperature (mT_s_) was calculated using the Ramanathan equation (Ramanathan, 1964). The parameter mean body temperature (mT_b_) was calculated using the formula of Sugenoya and Ogawa (1985): mT_b_ = (0.9.T_ty_ + 0.1 mT_s_).

### Blood Analysis

Blood was sampled before the first immersion (Pre) and immediately following the last 5-min break (Post) (Schematic 1). The blood samples were collected from the antecubital vein and transferred to serum-separating tubes. The samples were centrifuged at 3,000 rpm (2,000 × *g*) for 10 min at 4°C. Serum was then harvested and stored at −80°C until analysis. The cortisol level was determined using cortisol RIA CT (AMP, Germany) with γ-counter Cobra 5010 Quantum (Packard, Conroe, TX, United States). Irisin was determined using a commercial ELISA kit (Irisin EIA kit EK-067-16; Phoenix Pharmaceuticals, Burlingame, CA, United States) with a spectrophotometric reader. CK was measured with *N*-acetyl cysteine (NAC). LDH (U/L) was measured using the lactate-to-pyruvate method by Abbott ARCHITECT c16000 analyzer (Abbott Laboratories, Abbott Park, IL, United States).

### Statistical Analysis

Descriptive statistics are expressed as mean ± standard deviation (SD) using the commercially available computer software SPSS for Windows, version 21.0 (SPSS Inc., Chicago, IL, United States). Normality of the data were verified via Shapiro–Wilk normality test followed by calculating skewness and kurtosis of the statistics. Statistical significance was determined using a paired Student’s *t*-test to conduct a comparison between before (pre) and after (post) heat stimulation. Linear regression analysis and Pearson correlation coefficient were used to assess the correlations between variables. Significant differences were considered at *P* < 0.05.

## Results

### T_ty_ and mT_b_

**FIGURE 1:**
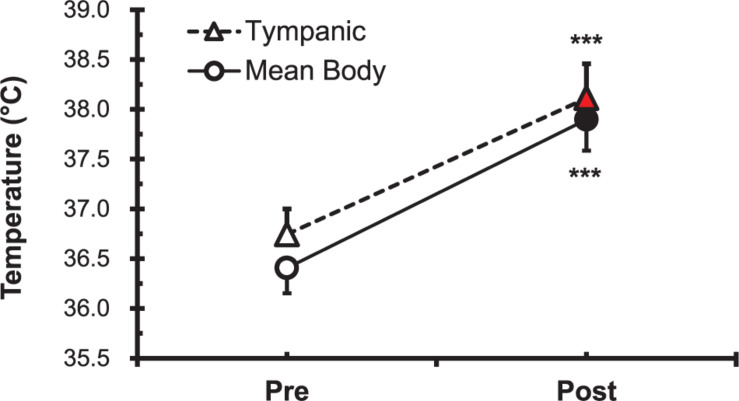
Comparison of tympanic temperature (T_ty_) and mean body temperature (mT_b_) before (Pre; ∘, △) and after (Post; •, ▲) hot water bath (42 ± 0.5°C) immersion. Values are mean ± SD. ***P < 0.001, statistically significant difference between Pre- and Post-exposure values.

The parameters T_ty_ and mT_b_ before (Pre) and after (Post) the heat stimulation are shown in Figure 1. Both T_ty_ and mT_b_ were increased significantly (*P* < 0.001) after immersion in the hot water bath (42 ± 0.5°C).

### Levels of Irisin

**FIGURE 2:**
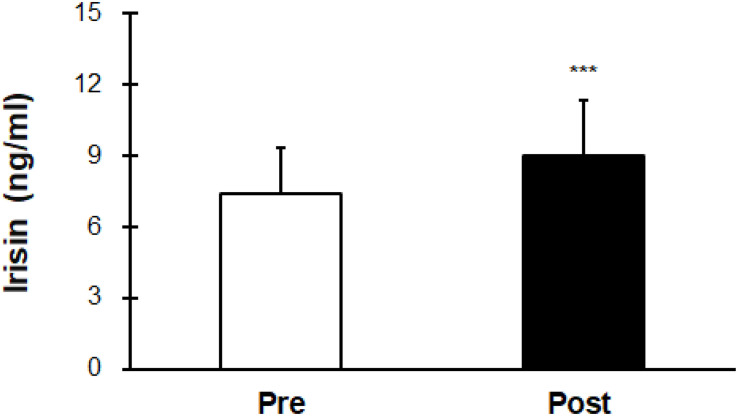
Comparison of serum irisin concentration before (Pre) and after (Post) hot water bath (42 ± 0.5°C) immersion. Values are mean ± SD. ***P < 0.001, statistically significant difference between Pre- and Post-exposure values.

**FIGURE 3:**
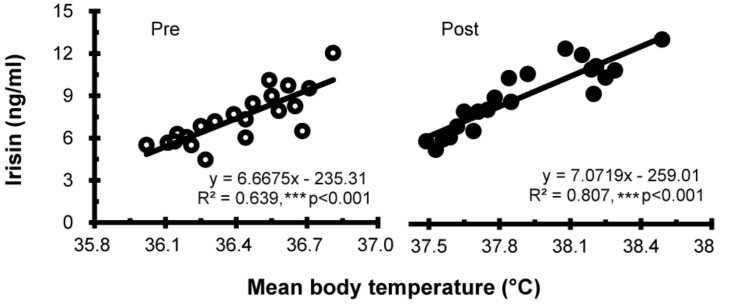
Correlation between the mean body temperature and irisin concentration before (Pre; ∘) and after (Post; •) hot water bath (42 ± 0.5°C) immersion. ***P < 0.001, statistically significant difference (Pre; *R*^2^ = 0.639, Post; *R*^2^ = 0.807).

Irisin levels were elevated after hot water immersion from 7.43 ± 1.88 to 9.00 ± 2.34 ng/mL (*P* < 0.001) (Figure 2). Correlation between mT_b_ and irisin before and after half-body immersion protocol is shown in Figure 3. A positive relationship between mT_b_ and irisin was observed before (*R*^2^ = 0.639, *P* < 0.001) and after (*R*^2^ = 0.807, *P* < 0.001) hot water treatment was also observed.

**FIGURE 4:**
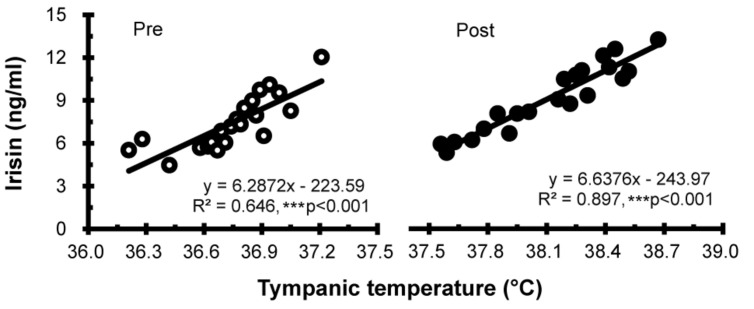
Correlation between tympanic temperature and irisin concentration before (Pre; ∘) and after (Post; •) hot water bath (42 ± 0.5°C) immersion. ***P < 0.001, statistically significant difference (Pre; *R*^2^ = 0.646, Post; *R*^2^ = 0.897).

Correlation between T_ty_ and irisin levels before and after half-body immersion protocol is shown in Figure 4. A positive relationship between T_ty_ and irisin was observed before (*R*^2^ = 0.646, *P* < 0.001) and after (*R*^2^ = 0.897, *P* < 0.001) hot water immersion was also observed.

### Levels of Cortisol, CK, and LDH

**FIGURE 5:**
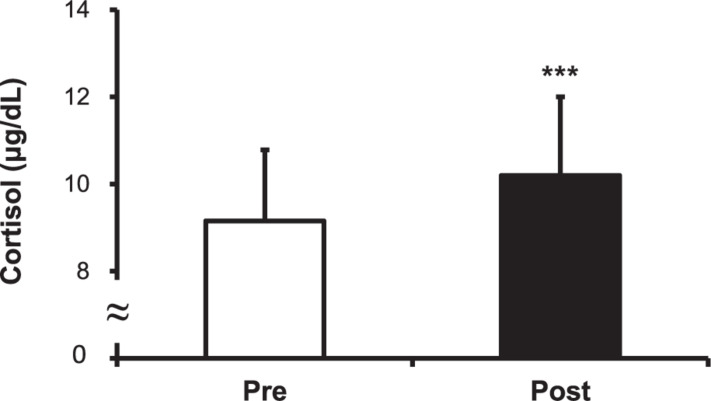
Comparison of serum cortisol concentration before (Pre) and after (Post) hot water bath (42 ± 0.5°C) immersion. Values are mean ± SD. ***P < 0.001, statistically significant difference between Pre- and Post-exposure values.

**FIGURE 6:**
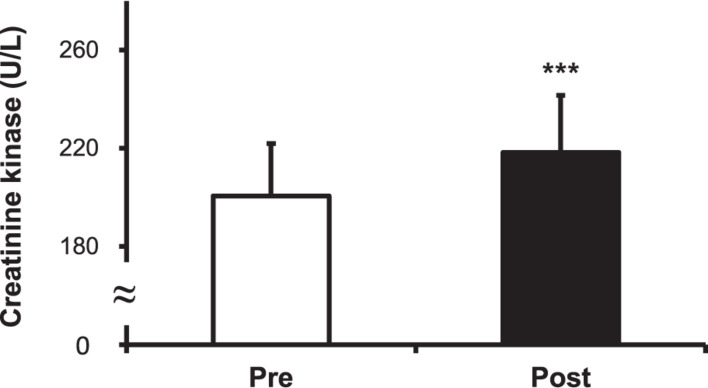
Comparison of serum creatine kinase (CK) concentration before (Pre) and after (Post) hot water bath (42 ± 0.5°C) immersion. Values are mean ± SD. ***P < 0.001, statistically significant difference between Pre- and Post-exposure values.

**FIGURE 7:**
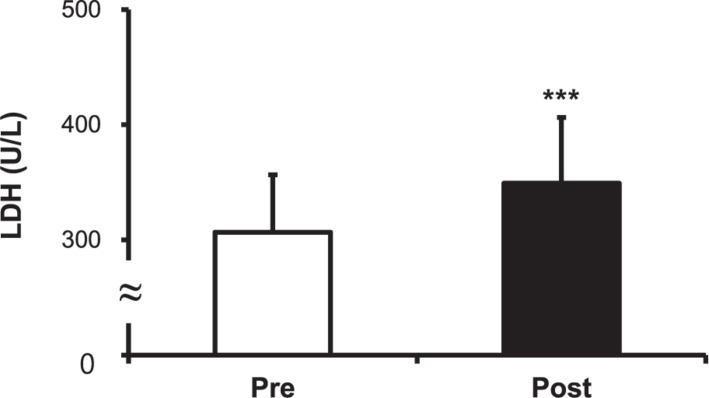
Comparison of serum lactate dehydrogenase (LDH) concentration before (Pre) and after (Post) hot water bath (42 ± 0.5°C). Values are mean ± SD. ***P < 0.001, statistically significant difference between Pre- and Post-exposure values.

The cortisol level increased after the hot water treatment, from 9.15 ± 1.63 to 10.20 ± 1.80 μg/dL (*P* < 0.001) (Figure 5). The CK level increased post-immersion in hot water bath, from 200.50 ± 21.43 to 218.33 ± 23.30 U/L (*P* < 0.001) (Figure 6). The LDH level increased after immersion in the hot water bath, from 306.67 ± 49.85 to 349.33 ± 57.07 U/L (*P* < 0.001) (Figure 7).

## Discussion

Irisin is believed to be secreted from the muscles in response to physical exercise and has beneficial effects (Bostrom et al., 2012). It has been recently considered as a potential therapeutic candidate due to its antioxidant activity against several diseases. Li et al. (2017) reported that an injection of irisin reduces ischemia-induced neuronal injury. Lu et al. (2015) demonstrated that systemic administration of irisin has a therapeutic effect against atherosclerotic vascular diseases in diabetics. Park et al. (2015) suggested that irisin facilitates the prevention of hepatic steatosis by attenuating oxidative stress. Batirel et al. (2014) also reported that irisin is a potential target in metabolic diseases such as non-alcoholic fatty liver disease. These diseases are related to oxidative stress, and irisin may act as an antioxidant.

The temperature of the human body is closely maintained at 37°C by regulating the balance between heat production and heat loss. Thermoregulation is centrally controlled by hypothalamus and peripherally via the sympathetic nervous system. And there are variable factors that could affect the thermoregulation such as circadian rhythm, climate acclimatation, hormones, and cytokines. Mild hyperthermia treatment has been shown to activate immunity against various diseases (Tomiyama-Miyaji et al., 2007). Thus, conventional hyperthermia including bathing in hot springs, a sauna, a home bath, or similar treatments might be effective in promoting good health (Tomiyama et al., 2015). However, a high temperature might also induce stress in healthy humans. Strong hyperthermia induces secretion of stress hormones (catecholamines and cortisol) (Won and Lin, 1995; Jimenez et al., 2007). Especially under stress, catecholamines, and cortisol are indicators of the status of autonomic nervous system and the hypothalamic–pituitary–adrenal axis.

In this study, we investigated the changes in serum irisin in response to heat stimulation-induced hyperthermia in humans. The body temperature, the level of the stress hormone cortisol, and the myokine irisin increased after heat stimulation. The positive relationships between the concentration of irisin and body temperature, became stronger after thermotherapy. Increased serum levels of other oxidative stress markers such as CK and LDH were also detected. We thus confirm that heat-induced oxidative stress resulted in an increase in circulating irisin, which also demonstrates that hyperthermia therapies such as immersion in warm water can induce beneficial effects similar to physical exercise.

After finding positive correlation between absolute data of the body temperatures and irisin concentration before and after half-body immersion, same analysis was conducted with their increments. Shifts of the T_ty_ and the irisin concentration showed positive, but not significant correlation. No other than positive trends without significance were found between the deltas of the temperatures, irisin concentration, and oxidative stress markers. It is expected that significant predictive models can be obtained by recruiting more subjects or by modifying the experiment protocol, such as varying the duration of heat loading or adding mid-time blood sampling. Oxidative stress and irisin concentrations were found to increase together after the passive heat loading, but the correlations between the amount of change was not significant. This may be due to a wide variety of remaining variables such as weight, muscle mass, sweating activity, and exercise habits affecting the change of body temperature and the amount of oxidative stress under the identical heat stimulation.

Because half-bathing in 42°C of hot water is a considerable burden on the human body, rests given between 5 min of immersion were essential. Taking a continuous bath in a lower, bearable temperature would require a substantial time to reach at the body temperature of 38°C.

Although the stop-and-go procedure and drink of lukewarm water have boggled the rise of temperature, our goal to increase the body temperature by passive heating was sufficiently achieved. Yet since there may be some differences between briefly repeated and continuous thermal stimulation, study comparing various heating protocols and diverse conducting media such as water, air, and infrared rays would be interesting.

The results of this study confirm the observations made by Samy et al. (2015) that the concentration of irisin was related to oxidative stress and muscle damage. Exposure to high levels of environmental temperature (e.g., 42 or 43°C) can trigger hyperthermia, hypotension, and cerebral ischemia (e.g., heatstroke) (Chang et al., 2007; Chen et al., 2013). Rats with heatstroke showed increased production of free radicals, higher lipid peroxidation, lower enzymatic antioxidant defenses, and higher enzymatic pro-oxidants in the brain (Chang et al., 2007; Chen et al., 2013). Increased levels of CK, LDH, and aspartate aminotransferase (AST) found in skeletal and cardiac muscle tissue are a reliable measure of the extent of tissue damage due to thermal injury during heatstroke (Francesconi and Mager, 1978; Alzeer et al., 1997). In the present study, heatstroke did not occur, but hyperthermia-induced oxidative stress certainly did. Although this is not a pathological change, serum CK, and LDH increased during hot water immersion, which means that heat stimulation induced damage to the skeletal and cardiac muscle tissues.

It has been suggested that heat stimulation intervention, such as sauna treatment and half-body immersion, has a wide range of health benefits and including alleviation of disease. Habitual hot baths have been shown to be associated with a reduced risk of developing cardiovascular, neurocognitive, and pulmonary diseases, as well as amelioration of chronic fatigue, chronic pain, arthritis, headache, and flu (Crinnion, 2011; Laukkanen et al., 2018). In addition, studies have shown that warming the body improves affective disorders and provides neuropsychiatric benefits (Rolls et al., 2008; Janssen et al., 2016).

The molecular mechanism of irisin up-regulation under heat stimulation, its antioxidative activity, quantification of therapeutic gain, and possible side effects need to be investigated before clinical application. However, as a simple indoor practice to alleviate various neuropsychiatric and general diseases, thermotherapy is worth to be investigated further.

## Data Availability Statement

The raw data supporting the conclusion of this article will be made available by the authors, without undue reservation.

## Ethics Statement

The studies involving human participants were reviewed and approved by Institutional Review Board on Human Subjects Research and Ethics Committees, Soonchunhyang University (No. 1040875-201611-BR-042). The patients/participants provided their written informed consent to participate in this study.

## Author Contributions

All authors listed have made a substantial, direct and intellectual contribution to the work, and approved it for publication.

## Conflict of Interest

The authors declare that the research was conducted in the absence of any commercial or financial relationships that could be construed as a potential conflict of interest.
